# The current landscape of optogenetics for the enhancement of adoptive T-cell therapy

**DOI:** 10.1093/discim/kyae019

**Published:** 2024-12-23

**Authors:** George R Smith, Max P Lee, Emma K Jennings, John R James

**Affiliations:** University of Warwick, Warwick Medical School, Coventry, UK; Present Address: University of Cambridge, Babraham Institute, Department of Immunology, Cambridge, UK; University of Warwick, Warwick Medical School, Coventry, UK; University of Warwick, Warwick Medical School, Coventry, UK; University of Warwick, Warwick Medical School, Coventry, UK

**Keywords:** adoptive T-cell therapy, optogenetics, CAR-T, eTCR-T, tumour-infiltrating lymphocytes, cancer, immunotherapy

## Abstract

Immunotherapy, the medicinal modulation of a host’s immune response to better combat a pathogen or disease, has transformed cancer treatments in recent decades. T-cells, an important component of the adaptive immune system, are further paramount for therapy success. Recent immunotherapeutic modalities have therefore more frequently targeted T-cells for cancer treatments and other pathologies and are termed adoptive T-cell (ATC) therapies. ATC therapies characterize various types of immunotherapies but predominantly fall into three established techniques: tumour-infiltrating lymphocyte, chimeric antigen receptor T-cell, and engineered T-cell receptor therapies. Despite promising clinical results, all ATC therapy types fall short in providing long-term sustained tumour clearance while being particularly ineffective against solid tumours, with substantial developments aiming to understand and prevent the typical drawbacks of ATC therapy. Optogenetics is a relatively recent development, incorporating light-sensitive protein domains into cells or tissues of interest to optically tune specific biological processes. Optogenetic manipulation of immunological functions is rapidly becoming an investigative tool in immunology, with light-sensitive systems now being used to optimize many cellular therapeutic modalities and ATC therapies. This review focuses on how optogenetic approaches are currently utilized to improve ATC therapy in clinical settings by deepening our understanding of the molecular rationale behind therapy success. Moreover, this review further critiques current immuno-optogenetic systems and speculates on the expansion of recent developments, enhancing current ATC-based therapeutic modalities to pave the way for clinical progress.

## Graphical Abstract

**Graphical Abstract:**
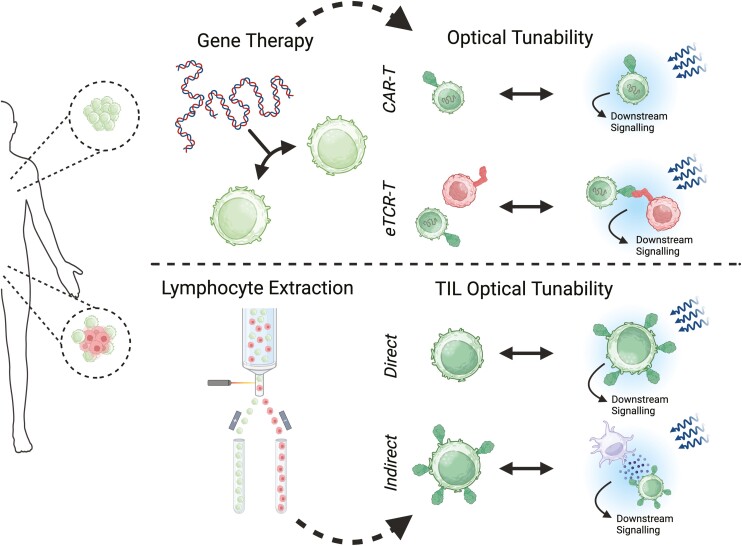

## Introducing immuno-oncology

Cancer is one of the leading causes of fatality worldwide [1]. It arises from unregulated cell proliferation and can manifest as a solid growing mass known as a malignant tumour with few effective treatments. Solid tumours that display sufficiently immunogenic antigen to break tolerance can be targeted naturally by the immune system, but evasion and dampening strategies evolved within the tumour microenvironment (TME) abrogate the efficacy of the immune system in combating cancer. Enhancing the immune response for a more robust defence against tumour growth was first attempted in 1891, with William Coley noting a reduction in tumour virulence if sarcoma patients were exposed to live attenuated *Streptococcus pyogenes* and *Serratia marcescens* [2]. Since then, harnessing the immune system to reduce cancer growth has grown into a diverse and well-funded field, known as immuno-oncology, with the collective goal of developing treatments that modify the host immune system to more effectively combat cancer. The landmark development of the hybridoma technique in 1975 enabled the selection and purification of monoclonal antibodies from immunized animals [3]. This paved the way for antibody-based drugs to become the first clinically applied immunotherapies for manipulation of the balance between activatory and inhibitory states in lymphocytes. In 1997, the Food and Drug Administration (FDA) approved the first therapeutic monoclonal antibody, Rituximab, with CD20 specificity for the treatment of non-Hodgkin’s lymphoma and there are now over 100 FDA-approved monoclonal antibodies against both cancerous and non-cancerous diseases [4].

**Figure 1.**
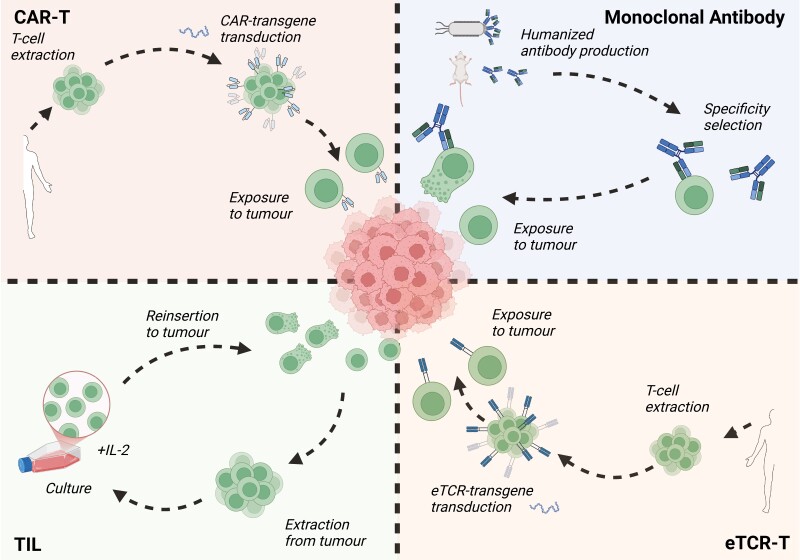
Classical methods of cancer immunotherapy. CAR-T therapy involves extraction of patient T-cells prior to transduction with a CAR-transgene specific for a cancer antigen. The T-cells, now with specificity to an antigen of interest, are reinserted into the patient to eliminate tumour cells expressing the antigen of interest. Monoclonal antibody therapy is often used to boost a host immune response against a tumour through either agonism of stimulatory signals provided by ligation to cell-surface receptors, or antagonism of inhibitors, such as the well-defined anti-Programmed Cell Death Protein-1 (PD-1) checkpoint blockade. TIL therapy extracts patient TILs, cultures them *ex vivo* to increase their population size and reinserts them to the tumour site for boosted tumour T-cell cytotoxicity. eTCR-T therapy is, in part, analogous to CAR-T therapy but with transduction of a transgene encoding a full TCR specific for an antigen rather than a chimeric receptor tethered to specific intracellular signalling domains.

Immune checkpoint inhibition (ICI) is one of the most characterized and successful uses of monoclonal antibodies, modulating (often inhibiting) receptor function to enhance immune anti-cancer response, and is based on early work from Jim Allison and Tasuku Honjo, who shared the 2018 Nobel prize for their development [5–7] (Fig. 1). Clinical ICI results are generally positive, with multiple clinical studies citing an improvement in durable response rates when solely compared to currently applied chemotherapies [8, 9]. However, despite undoubtable success, questions remain over the efficacy of ICI therapy on overall or progression-free survival when compared to chemotherapy and suggest its effects of increased response rate do not correlate with survival, though this conclusion is seemingly cancer- and age-dependent [10, 11]. Drawbacks to monoclonal antibody-based therapies are well-known. The most obvious shortcoming is poor penetration into solid tumours, with Beckman *et al.* citing only ~20% of administered antibodies as interacting within the target tumour [12, 13]. In addition, when used to enhance an immune response against a specific antigen through agonism or antagonism of co-stimulatory or co-inhibitory receptors, respectively, there is little evidence of *in vivo* complement cascade triggering and many monoclonal antibodies even appear to elicit an inhibitory response when bound to activatory immune-cell receptors [14]. The overall clinical efficacy of monoclonal antibodies is therefore up for debate. These limitations, together with frequent side effects and substantial resources and time required for their generation, meant that a more robust initiator of an immune response was required. However, numerous breakthroughs in the immuno-oncology field have occurred to improve monoclonal antibodies such as cancer vaccines, cytokine treatments, and monoclonal antibody enhancements such as bispecifics and nanobodies. Yet, the most notable and now widely researched success of immuno-oncology has occurred through the redirection of T-cells to antigens of interest through adoptive T-cell therapy (ATC).

## Adoptive T-cell therapy

### Tumour-infiltrating lymphocytes

ATC involves modifying T-cells to direct an immune response against a specific target (Fig. 1).

The field of ATC began in 1863 with Rudolf Virchow’s discovery of tumour-infiltrating lymphocytes (TILs), in which he observed lymphoid cells within abnormal tissue growths [15]. TILs have since been defined as mononuclear T-lymphocytes present within the TME and thus deemed as T-cells expanded to combat tumours. Persistent research has subsequently been undertaken to understand both how these T-cells are different from their non-TIL counterparts and whether their anti-cancer capabilities can be enhanced as a tumour treatment [16, 17]. It took nearly a century after initial discovery for TILs to be isolated and clinically applied, with successful isolation, and thus the genesis of the ATC field, published by Steven Rosenberg in 1986 [18].

Despite claiming that TILs were 50 to 100 times more effective in therapeutic potency than non-tumour-infiltrating activated lymphocytes, evasion, and dampening strategies within the TME severely attenuated TIL efficacy [19]. To counter this, Rosenberg hypothesized that increasing the number of TILs at the tumour site could amplify the anti-tumour immune response, potentially reducing tumour growth rate or even shrinking tumour size [18]. His method involved extracting tumour tissue, mincing, and then using agitation and connective tissue cleavage enzymes to break the tumour down into a single-cell solution. The solution containing both TILs and tumour cells was incubated with Interleukin (IL)-2 (a T-cell growth factor) for several weeks to expand the TIL population while the tumour population was depleted. Once enough TILs were obtained with negligible cancer cell numbers, Rosenberg re-infused them into mice with induced pulmonary and hepatic metastases [18]. Results seen were promising, with 50–100 times less TILs needed than lymphokine-activated killers to mediate cancer regression. Co-administration with the chemotherapy cyclophosphamide even demonstrated regression in the size of large tumours.

However, due to the inaccessibility of tumours away from the skin and potent immune-dampening strategies used by cancers, it was clear TILs would not become a comprehensive cancer treatment. However, if T-cells could be isolated at sites away from the tumour and engineered to become cancer specific with potential mechanisms to prevent suppressive effects in the TME, issues faced by TILs could be bypassed with more effective clinical therapy.

### Chimeric antigen receptor T-cell therapy

The generation of Chimeric antigen receptor-T (CAR-T) cells in 1993 by Zelig Eshhar provided an answer to the issues found with TILs [20]. Eshhar engineered a chimeric ‘T-body’ composed of a single-chain antibody fragment (scFv) to provide antigen specificity, linked to the intracellular signalling domains from the T-cell antigen receptor to facilitate T-cell activation. Upon binding to the target antigen, as specified by the extracellular binding moiety, the CAR-expressing T-cell is activated in a major histocompatability complex (MHC)-unrestricted manner (Fig. 1).

The first generation of CAR-T cells did not have a significant clinical impact due to issues with low persistence, signal transduction efficiency, and cytotoxic potency [21, 22]. Nonetheless, CAR-T cells demonstrated the feasibility of fusing a binding site of interest to a T-cell, thereby conferring an immunological response to a target antigen without MHC restriction. Following this, the field of CAR-T cells vastly expanded, with three more generations developed. The second generation contained an extra co-stimulatory domain and was the first CAR-T type to be applied clinically [23]. Tisagenlecleucel, with *α*-cluster of differntiation (CD)19 specificity, was the first CAR-T cell that received FDA approval for the treatment of B-cell leukaemias and has displayed remarkable results, particularly in patients under 23 years old with B-cell acute lymphoblastic leukaemia, where it achieved an 81% remission rate within 3 months and persistence within the patient at 20 months [24].

There are currently six FDA-approved CAR-T cells used for the treatment of B-cell lymphomas and multiple myeloma, which all utilize the second generation CAR format [ 25]. Despite the undeniable success of second generation CAR-T cells, problems remain with suboptimal signal transduction efficiency, persistence, cost, and highly toxic side effects including cytokine release syndrome (CRS) and immune-cell induced neurotoxicity syndrome. Additional co-stimulatory domains were incorporated into second generation CARs to form their third generation, with the most promising being CD3*ζ*-CD28-41BBbased [25]. However, despite the sporadic mention of third-generation CAR-T cells as possessing more efficient signal transduction with potent anti-tumour effects in murine models [26], third-generation CAR-Ts displayed little difference when used in human clinical trials and even seemed to present worse side effects and reduced persistence [27, 28].

Fourth-generation CAR-T cells encompass the complete complex of a second-generation CAR-T cell but with an additional nuclear factor of the activated T-cell (NFAT)-responsive transcription factor cassette. This modification enhances the CAR-T cell with the ability to induce cytokine secretion upon binding of the CAR to its cognate antigen, creating a more permissive TME for improved persistence and activation of proximal innate immune cells to enhance the anti-tumour response. Pre-clinical tests show notable efficacy of fourth-generation CAR-T cells, displaying effective homing and activation of nearby innate immune cells, dampening of the adverse conditions with the TME and even killing of antigen-loss tumour cells with IL-12 releasing CAR-T cells [29, 30]. The first clinical trial testing fourth-generation CAR-T cells with combinations of IL-12, IL7, and CCL9 secreting domain cassettes for the treatment of Nectin4/Fibroblast Activation Protein (FAP) + malignant solid tumours is currently underway [31].

### Engineered T-cell receptor therapy

Despite incorporating co-stimulatory and cytokine-inducing domains to intracellular signalling regions, the reduced efficiency of signal transduction from a CAR, compared to the endogenous T-cell Receptor (TCR), poses a significant drawback to the scale of immune response mounted after binding to cognate antigen. Subsequent work in 2005 showed that despite being MHC restricted, engineering a native TCR (eTCR) to a specific antigen could not only result in a more natural and potent immune response than CAR-T therapy but also confer improved antigen sensitivity and persistence [32]. This therapy is subsequently termed eTCR-T therapy (Fig. 1). The eTCRs for specific pMHC can be incorporated into T cells analogously to CAR expression in CAR-T therapy. The FDA approved the first eTCR-based therapy, Tecelra, on an accelerated approval process in August 2024. Tecelra targets melanoma-associated antigen 4, a common tumour-associated antigen (TAA) for metastatic synovial sarcoma [33]. As eTCR-T therapy can recognize intracellular and transmembrane tumour antigens, it possess a wider scope of potential targets than CAR-T therapy. There are therefore many eTCR-T cell therapies in the first and second stages of clinical trials, with 22 total clinical trials of eTCR therapies having been extensively evaluated [34]. In each case, eTCR therapies failed clinical approval due to high cost, strict MHC restriction, and lack of tumour antigen specificity.

Methods to improve the effectiveness of eTCR therapy are rapidly increasing. One particularly interesting technique is the use of ‘rejuvenated’ induced pluripotent stem cells from antigen-specific T cells (T-iPSCs) [35]. Here, induced pluripotent stem cell T cells (T-iPSCs) are transduced with an antigen-specific TCR to differentiate into CD8 + antigen-specific T-cells as a lower cost, quicker, and more effective method of introducing TCR-specificity than transducing terminally differentiated peripheral CD8 + T-cells. These T-iPSCs further display comparable anti-tumour capabilities to peripheral T-cells but can theoretically be continuously applied to multiple patients from a single product if Human Leukocyte Antigen (HLA)-matched and quality controlled. It is further thought that maintaining a memory-like eTCR phenotype could increase persistence and potentially effectiveness against solid tumours. Multiple methods have been hypothesized, but none have yet cited substantial *in vivo* improvement in persistence or notable effectiveness.

ATC is a progressive and frontline sub-section of modern immunotherapy with ample active research. Yet significant drawbacks such as poor tumour infiltration, low activation efficiency, and high costs yield clinical ATC applications as yet to be complete [36, 37]. These issues are yet to be solved, in part, due to an incomplete understanding of T-cell signalling and the biophysical constraints of receptor antigen binding. Understanding these mechanisms are paramount for realizing the clinical potential of different ATCs yet many immunotherapies are developed without fully understanding the mechanisms by which they work, limiting the effectiveness of troubleshooting drawbacks when applied in clinic. There have been numerous technological developments that have progressed immuno-oncological understanding with this review primarily focussing on optogenetics as a method for both single-cell and population-level investigation of the immunological anti-cancer response.

### Investigating ATC with optogenetics

Using optogenetics as an experimental tool is a relatively recent phenomenon in biology; it involves coupling light-sensitive proteins to biological agents of interest to provide spatio-temporal control over protein function in response to light. One of the earliest demonstrations of light-dependent activation was reported by Miesenböck *et al*. who used genetic ‘ChARGing’ to co-express Drosophila rhodopsin in neurons, making them sensitive to light [38]. Optogenetic control over ion channel function has since become a widely used tool in neurobiology. The application of optogenetics to immune-cell function is more recent and has emerged as a valuable tool for studying processes such as cytokine release, T-cell receptor (TCR) signal transduction, and the kinetics of ligand-dependent activation. Optogenetics is also starting to make a clinical impact, particularly in the development of immunotherapies, leading to the multidisciplinary field of opto-immunoengineering [39], with the primary goal of enhancing immunotherapies for treating cancer and other diseases.

### Optogenetics in CAR-T cells

As previously discussed, CAR-T therapy is a revolutionary immunotherapy that mounts an immune response against an antigen of interest independent of MHC presentation. Optogenetic manipulation has thus been utilized to optically tune activation of CAR-T cells at different levels of signal transduction from receptor binding to gene transcription. This is done with the aim of both understanding the biophysical parameters of CAR-T, and thus T-cell, signalling and inform further optimization of CAR-T therapy to improve upon the current drawbacks in persistence, cytotoxic potency, and antigen specificity.

**Figure 2.**
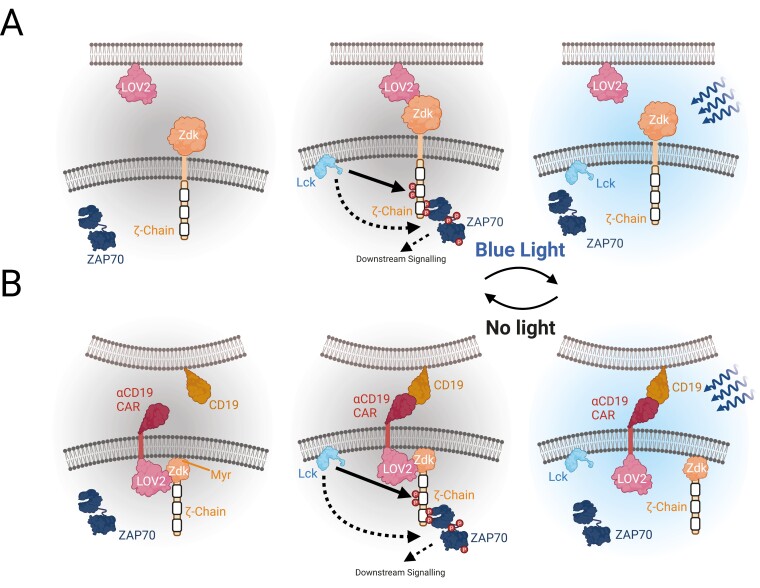
Intracellular and extracellular methods of investigating CAR-T cell signalling with optogenetics. Optogenetic investigation of CAR-T cells can be used (A) at the extracellular interface to optically tune receptor-ligand binding [40] or (B) to periodically couple and decouple intracellular signalling domains from ligand-engaged receptors [41].

One of the first studies using an optogenetic system within a CAR came from the Weiner lab, to explore the role of kinetic proofreading on T-cell peptide discrimination (Fig. 2A) [40]. Here, the light-oxygen-voltage-sensing domain 2 (LOV2) trap and release of protein (LOVTRAP) system was used that contains two functional units: LOV2, a photosensor domain from *Avena sativa* phototrophin1 and ZDark (Zdk), a small domain that was designed by directed evolution to bind LOV2. In the dark, these two domains are tightly bound, but upon blue light illumination (400–500 nm), LOV2 alters conformation to prevent Zdk association. Tischer and Weiner [40] used Zdk in place of an antigen-recognizing scFv region to confer specificity of the CAR to LOV2, which was presented on a supported lipid bilayer. By decreasing light intensity to vary ligand binding half-lives on T-cell signalling, they observed a strong correlation between the duration of ligand binding and the potency of T-cell signalling. These results aid research into the previously disputed field of how T-cells differentiate between self and non-self peptide-bearing MHC (pMHC), demonstrating that small changes in ligand half-life are likely the defining factor.

The LOVTRAP system is an attractive investigative tool as it allows the association and dissociation of LOV2 and Zdk to occur in a matter of seconds [42]. It has also been used to investigate CAR-T exhaustion by enabling pulsatile CAR-T activation. Exhaustion describes the irreversible reduction of CAR-T effector function in response to persistent signalling and is a main cause of treatment failure in the clinic. With constant activation of CAR-T cells rapidly inducing exhaustion, pulsatile signalling of CAR-T cells is a theory hypothesized to limit its onset. One method to achieve pulsatile stimulation is to periodically decouple intracellular signalling from the extracellular binding moiety of a clinically relevant *α*-CD19 CAR, which was the approach taken by James *et al*. [41] (Fig. 2B). This work showed that a reduction in the duty cycle (total time a CAR-T cell is active over a set period) can potentially minimize CAR-T cell exhaustion through pulsed periods of activation. Research by O’Donoghue *et al.* who used a blue light-dependent optogenetic system to mediate CAR-T activation also shows that pulsed signalling potently activates *α*-CD19 CAR-T cells that are sensitive to activatory signals on a minute timescale [43]. As constant signalling is a key component of inducing exhaustion, pulsed signalling of CAR-T cells at the right frequency may be an activation mechanism that retains effector function for longer periods.

**Figure 3.**
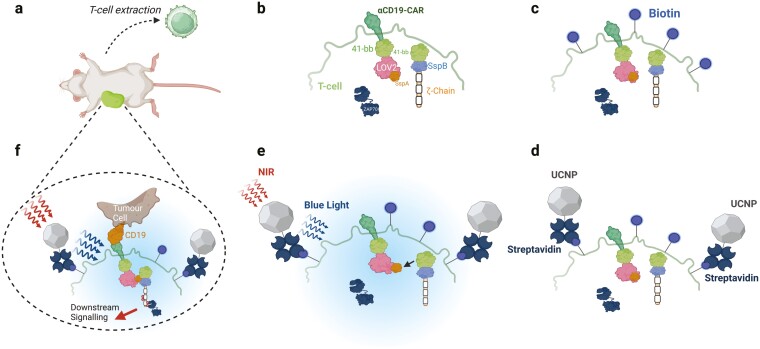
Method for clinical application of optically tunable CAR-T cells. Nguyen *et al.* [44] described a method of clinically applying LiCARs for fewer non-target or extraneous effects than conventional CAR-T therapy. T-cells were extracted from mice (a) and transduced with CAR domains containing optical tunability through the iLID system to form LiCARs (b). The LiCARs were then biotinylated (c) for binding to streptavidin coated UCNPs (d). Once bound to the LiCAR, the UCNPs were exposed to NIR radiation that is converted into blue light, subsequently activating the LiCAR through coupling the extracellular binding moiety and intracellular signalling domain (e). CAR-T cells, when administered *in vivo*, were only activated at areas of NIR exposure and in the presence of the specific tumour ligand (f).

While the optogenetic studies described above are focussed on discovery-based research, some clinical use of light-tunable CAR-T cells has been attempted (Fig. 3) [44]. Clinical application of photo-inducible components is made more challenging by the significant absorption and scattering of light as it penetrates through tissue *in vivo* [45]. Many optogenetics tools are derived from plant photoreactive proteins that require blue light (~450 nm) excitation, which renders direct optogenetic excitation highly improbable for any application below the skin’s surface [46]. To overcome this, Nguyen *et al.* created a photoreactive CAR-T cell that used upconversion nanoparticles (UCNP) that increase the frequency of incident light, in this case from near infrared (NIR) that can penetrate deeply into the body, to blue light [44]. In this study, light-switchable CAR-T cells, termed LiCARs, were tested for their ability to induce an anti-tumour response dependent on both antigen binding and exposure to blue light. These LiCARs were used in mice through the combination of UCNPs and antigen availability for CAR-T activation. This technology allows for control of CAR-T cell-mediated cytotoxicity against CD19-bearing cells deep within tissue, since NIR is far less absorbed by haemoglobin or body fluids than emission within the visible spectrum [45]. Light tunability was achieved by splitting the CAR complex into two parts as before (Fig. 2B) but utilizing the improved light-induced dimerization (iLID) system [46] in place of LOVTRAP. *In vivo* application of this system in mice showed that deep-tissue-penetrable NIR light can effectively activate *α*-CD19 LiCARs for the specific destruction of CD19 + melanoma cells. In addition, B-cell aplasia was non-existent in LiCAR-treated mice, but prevalent in wildtype (WT) CAR-T-treated mice and when a well-established xenograft model of CRS was applied, mice treated with LiCARs displayed an extraordinarily reduced onset of both weight-loss and IL-6 release. Despite also being performed in mice, with penetration of NIR light in humans likely requiring substantially enhanced and likely intravascular delivery methods, these results show that optogenetic manipulation of CAR-T cells is not simply a proof-of-concept enabling technology, but can be effectively utilized as a method for improving the safety and effectiveness of CAR-T therapy.

Whilst NIR light has been shown to penetrate up to 10 cm into tissue, it can not pass through bone, limiting the depth and therefore widespread use of light-dependent CAR-Ts [47]. Endovascular therapy-based light illumination technology was a potential solution to this issue and uses the physical insertion of an optical diffuser within a transparent catheter to allow light penetration throughout the body [48]. Whether this would generate enough energy to trigger a substantial response from the UCNP for activation of LiCARs is unclear, and would only work when targeting tissues proximal to the vascular lumen. Therefore, while the possibility of clinical LiCAR application is very intriguing, it is unlikely to be a generalized method for cancer treatment until methods of light penetration for better system coverage are established.

Multiple investigations have employed optogenetics as a method to periodically activate T-cells, thus delineating biophysical mechanisms behind complete T-cell activation [40, 41, 44]. The studies mentioned all claim that pulsed signalling effectively activates T cells with a chimeric receptor, and in some cases, more potently than constant stimulation. Neither studies, however, mention the effects of pulse signalling on inhibitory markers. It is possible that the potentially enhanced anti-tumour effects of CAR-T cells exposed to pulsatile signalling are due to differentiation to a more robust cell type that can maintain activation without checkpoint inhibition resulting in exhaustion. It would therefore be interesting, through using the optogenetic systems described, as manual tunability tools, to see whether pulsatile signalling alters the inhibitory marker signature or activity of Immunoreceptor Tyrosine-based Inhibition Motifs. If so, pulsatile signalling of CAR-T cells with an incorporated optogenetic system might be a viable method for improved *in vivo* expansion of CAR-T cells for differentiation into a more effective therapeutic agent against solid tumours.

### Optogenetics in engineered TCR-T cells

CAR-T cells currently dominate the field of ATC due to the variability in antigen specificity and the range of potential targets. However, shortcomings in recognition of intracellular antigen and low persistence have continued research into eTCR-T-cell therapy for a more native T-cell-like response in the treatment of solid tumours [49, 50]. Optogenetics has therefore been a prevelant method of investigating eTCR-T cell dynamics for both proof-of-concept and clinical development.

**Figure 4.**
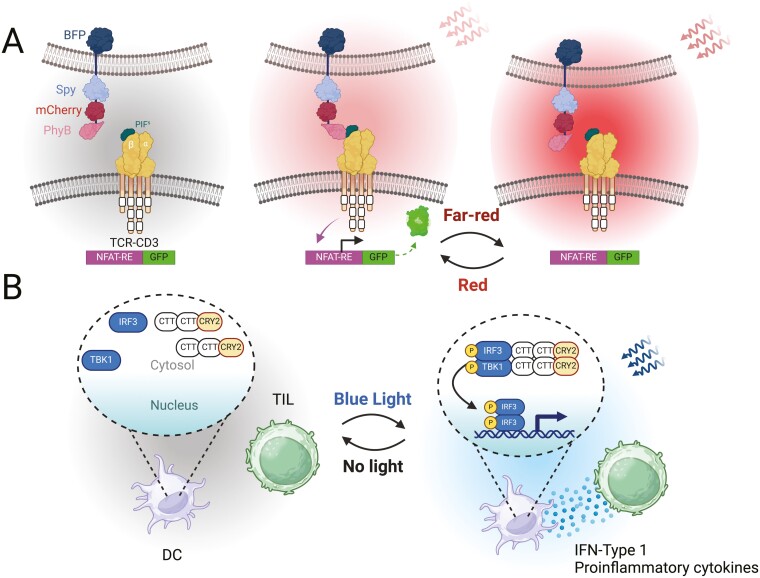
Optogenetic experiments with eTCR-T cells and TILs. A) An investigation by Idstein *et al.* [51] who used eTCR-Ts to periodically bind an APC presenting photo-sensitive domain PhyB. Upon red light illumination, PhyB would bind to the eTCR at the PIFs region. If exposed to far-red illumination, this interaction would cease in a reversible manner. B) Dou *et al.* [52] mimicked the effects of the cyclic GMP-AMP synthase-stimulator of interferon genes (cGAS-STING) pathway in bone marrow dendritic cells (BMDCs) tethering the STING-cargo to cryptochrome regulator 2 (CRY2) rather than STING. Upon blue light exposure, the CRY2 components cluster to induce phosphorylation and nuclear localization of cargo, analogous to STING clustering in response to cyclic guanosine monophosphate–adenosine monophosphate (cGAMP). The result is induced interferon regulatory factor 3 (IRF3) transcription and subsequent IFN-type 1 and proinflammatory cytokine release with blue light dependence through LISmores. These cytokines could therefore induce TILs to become more robust and persistent in the TME.

As eTCR-T cells are analogous to standard T-cells with specific receptor recognition, advancements in eTCR-T therapy often come from an increasing understanding of native TCR signalling. However, in the field of optogenetics, it is commonly eTCR-T cells that are used to delineate facts about T-cell signalling due to the requirement for a specific receptor to either bind to a ligand with light-dependent presentation or to harbour optical tunability itself. In this regard, optical tunability of a modified TCR was achieved by Schamel *et al.* using the PhyB/PhyB interacting factor (PIF) optogenetic system (Fig. 4A) [51]. Red light illumination causes PhyB domain to interact with PIF, which is fused to the *β* chain of TCR; infrared illumination reverses this association with the PhyB-expressing cell. This investigation, like those described in CAR-Ts, provided oscillatory stimulus through ligand binding and observed that NFAT signalling persisted for over 8 min of no ligand binding but was attenuated without binding for 30˜ min.

Jaeger *et al.* investigated the effects of light-dependent pulsatile signalling of T-cell engagers. T-cell engagers do not directly manipulate intracellular T-cell machinery, but rather the exposure of T-cells to their cognate antigen to generate a more natural T-cell response. Jaeger *et al.* achieved this through periodically exposing TCR on T-cells to antigen through a recombinant T-cell engager termed the Light-inducible T cell Engager (LiTE) [53]. This system similarly makes use of the PhyB/PIF system, where PIF is now fused to a mAb targeting the mouse TCR to form the LiTE region. The LiTE region therefore binds mouse TCR to LiTE-TCR complexes. If the PhyB is exposed to the light of 656 nm, it has an open confirmation where LiTE-TCRs can bind and cause TCR immobilization and accumulation, triggering downstream signalling. If the system is exposed to the light of 730–760 nm light then the PhyB-LiTE interaction is abrogated, allowing tunability of TCR signalling. The investigation further suggested that pulsatile activation of T-cells polarized them to a T-helper type 2 phenotype with reduced effector functions, but with activation times all lower than an hour, it is unlikely T-cells had time to undergo such differentiation and the results seen are simply just through constant low levels of signalling. The investigation does however provide an interesting proof-of-concept where T-cells can be attuned for specific activation and inactivation times. Whilst this is also not a standard eTCR investigation, the results can be used to infer that pulsing can be achieved with a native TCR rather than just the previously discussed CAR-T cells. Furthermore, if it is the case that pulsatile signalling can streamline T-cell differentiation within an hour, then this could be a viable method for directed polarization of eTCR-T cells and CAR-T cells to a more persistent cell type before insertion into the tumour environment.

### Optogenetics for the optimization of TILs

TILs are the most native form of ATC as they are isolated and expanded, generally not requiring genetic manipulation or physical alteration before being reinserted back into a patient. Optogenetic studies of TILs are therefore commonly used for investigating T-cell mechanics within tumours rather than for optimization of TIL therapy.

TILs were created with the rationale that the TME can overcome the efficacy of the natural anti-tumour response, whereby enhanced numbers of TILs could potentially attenuate these effects through increasing the number of T-cells penetrating deep into the tumour [18]. Substantial dampening of TIL activity is, however, still seen as one of the most prominent drawbacks of TIL therapy [54]. There is therefore considerable research being undertaken to improve the conditions within the TME to make them more TIL permissive. Dou *et al.* aimed to do this in 2023 by incorporating optogenetic tunability of cytokine release in proximal dendritic cells to provide cytokine-dependent signals to the TILs that will maximize persistence and therefore increase deep-tumour penetration of T-cells (Fig. 4B) [52]. Dou *et al.* chose the cGAS-STING pathway, responsible for the detection of pathogenic cytosolic DNA and the subsequent release of type-1 interferon and proinflammatory cytokines. It was manipulated through the incorporation of light-inducible SMOC-like repeats (LISmore) in dendritic cells. These LISmores enable *in vivo* spatio-temporal control of STING-like signalling through mimicry of type-1 interferon (IFN) production pathway. Blue light irradiation of LISmore transduced cells displayed increased release of proinflammatory cytokines, IFN-*α*, and IFN-*β* as well as enhanced dendritic cell maturation than wild types. These results ultimately lead to improved efficiency of antigen TAA presentation, more effectively activating CD8 + TILs, observed through upregulation of IFN-*γ*, Ki67n, and CD69, that displayed enhanced anti-tumour effects against subcutaneously injected melanoma cells in a murine model.

As well as depleting immunological activation signals, as shown by Dou *et al.*, the TME also generates substantial metabolic competition between TILs and tumour cells. This is done via a plethora of mechanisms including glucose deprivation, hypoxia, and the creation of metabolic intermediates such as tryptophan and kynurenine all with the rationale of dampening the potency of potential anti-cancer lymphocytes within the TME, obviously limiting the success of TIL therapy. Methods have been tested, with some success, to combat the adverse metabolic environment including culturing T-cells in a hypoxic environment to enhance a T-cell population less reliant on oxygen, proviral Integration site for Moloney leukemia virus (PIM) kinase inhibition to improve glucose uptake efficiency, and phosphoenolpyruvate carboxykinase-1 overexpression for increased phosphoenolpyruvate production that enhances effector function of T cells. In 2018, Amitrano *et al.* aimed to improve the metabolic conditions in the TME optogenetically by creating a genetically encoded light-activated proton pump embedded within the mitochondrial inner membrane termed OptoMit-On [55]. OptoMit-On further enhanced mitochondrial adenosine triphosphate (ATP) production of activated CD8 + T-cells from mice and provided a method for increasing the rate of oxidative phosphorylation through light illumination. CD8 + cells with enhanced ATP production further displayed increased migration and granzyme B expression. This investigation showed that manipulating ATP production can increase the levels of oxidative phosphorylation in T-cells and provides an avenue through which TILs can be enhanced by increasing ATP production or through other methods of enhancing the rate of oxidative phosphorylation.

Two differing methods of enhancing TIL therapy through optogenetic methods have been described, one primarily utilizing a signalling-based approach and one through enhancing lymphocyte metabolic efficiency. Both methods described considerable success in enhancing the effector function of T-cells and it would be interesting to observe the hybrid effect of both avenues in one optogenetic investigation. Despite this, it is unlikely these methods will be applied *in vivo* due to restrictions in deep penetration of activating light, especially in the TME. It is possible for the use of UCNPs, as described earlier, to convert NIR irradiation into blue light but it seems more conceivable that these methods may either inform an improved gene-based therapy for the production of TME-resistant lymphocytes or provide insight into the optimal environment for culturing of TILs before *in vivo* insertion where optogenetic stimulation is more feasible.

## Summary

Optogenetics is becoming an established tool for studying ATCs due to its reversible activation, rapid stimulus reaction times, and nominal influence on other cellular processes. Light-mediated control has further provided a means to specifically observe mechanisms of T-cell activation for the subsequent optimizing of current ATCs. Nevertheless, the full application of optogenetics to ATC therapy has yet to be fully realized.

CAR-T cells currently dominate the space of optogenetics in adoptive T-cell therapy. There are multiple ways of integrating optical tunability into CAR-T cells and are seen as the model of choice due to the reductionist methods of activation, allowing precise identification of key components of the signalling cascade. Furthermore, optogenetic CAR-T cells also enhance understanding of TCR-based T-cell activation and allow inference into improvements to both TIL and eTCR-T therapy. There is, however, room for expanded research with eTCR-T cells as they display a more natural response that better resembles wild-type T-cells. It would therefore be interesting to observe whether the optogenetic space in eTCR-T research will incorporate the range of optogenetic systems as seen within CAR-T research. Not only would this vastly improve current knowledge of T-cell signalling, eTCR-therapy outcome, and potential enhancement to TILs, but it would also enable the identification of vital signalling components of T-cells that can be incorporated into CAR-T cells to make them more efficient signal transducers.

## Data Availability

The authors confirm that the data supporting the findings of this study are available within the article [and/or] its supplementary materials.
